# Analysis of the Leukocyte Response in Calves Suffered from *Mycoplasma bovis* Pneumonia

**DOI:** 10.3390/pathogens9050407

**Published:** 2020-05-24

**Authors:** Katarzyna Dudek, Dariusz Bednarek, Urszula Lisiecka, Anna Kycko, Michał Reichert, Krzysztof Kostro, Stanisław Winiarczyk

**Affiliations:** 1 Department of Cattle and Sheep Diseases, National Veterinary Research Institute, 57 Partyzantów Avenue, 24-100 Pulawy, Poland; dbednarek@piwet.pulawy.pl; 2 Department of Epizootiology and Clinic of Infectious Diseases, Faculty of Veterinary Medicine, University of Life Sciences in Lublin, 30 Głęboka Street, 20-612 Lublin, Poland; urszula.lisiecka@up.lublin.pl (U.L.); genp53@interia.pl (S.W.); 3 Department of Pathology, National Veterinary Research Institute, 57 Partyzantów Avenue, 24-100 Pulawy, Poland; anna.kycko@piwet.pulawy.pl (A.K.); reichert@piwet.pulawy.pl (M.R.)

**Keywords:** *Mycoplasma bovis*, cattle, leukocytes, phagocytosis, oxygen metabolism

## Abstract

*Mycoplasma bovis* is known to be a cause of chronic pneumonia in cattle. To date, the disease pathomechanism has not been fully elucidated. Leukocytes play a key role in host antimicrobial defense mechanisms. Many in vitro studies of the effect of *Mycoplasma bovis* (*M. bovis*) on leukocytes have been performed, but it is difficult to apply these results to in vivo conditions. Additionally, only a few studies on a local immune response in *M. bovis* pneumonia have been undertaken. In this study, the experimental calf-infection model was used to determine the effect of field *M. bovis* strains on changes of the peripheral blood leukocyte response, including phagocytic activity and oxygen metabolism by cytometry analyses. An additional aim was to evaluate the lung local immunity of the experimentally infected calves using immunohistochemical staining. The general stimulation of phagocytic and killing activity of peripheral blood leukocytes in response to the *M. bovis* infection points to upregulation of cellular antimicrobial mechanisms. The local immune response in the infected lungs was characterized by the T- and B-cell stimulation, however, most seen in the increased T lymphocyte response. Post-infection, strong expression of the antigen-presenting cells and phagocytes also confirmed the activation of lung local immunity. In this study—despite the stimulation—both the peripheral and local cellular antimicrobial mechanisms seem to appear ineffective in eliminating *M. bovis* from the host and preventing the specific lung lesions, indicating an ability of the pathogen to avoid the host immune response in the *M. bovis* pneumonia.

## 1. Introduction

*Mycoplasma bovis* causes many disorders in cattle, such as pneumonia, arthritis, mastitis and keratoconjunctivitis, from which chronic pneumonia is one of the most diagnosed [1,2,3]. To date, the pathomechanism of *M. bovis* pneumonia has not been fully elucidated. One such mechanism is the ability of the pathogen to modulate the host immune response [4]. It has been previously confirmed that *M. bovis* possesses both immunostimulating and immunosuppressive properties, most demonstrated in vitro studies. *M. bovis* can induce strong TNF-α responses in the exposed macrophages isolated from mycoplasma-free bronchoalveolar lavages of adult cattle [5]. The ability of *M. bovis* to modulate different neutrophil functions has been demonstrated by Jimbo et al. [6]. After incubation of *M. bovis* with neutrophils isolated from clinically healthy animals the induction of the cell apoptosis and increased elastase production was observed. The same study showed upregulation of pro-inflammatory cytokines—i.e., TNF-α and IL-12—but with no effect on TGF-β production [6]. Otherwise, it was revealed that *M. bovis* can inhibit the oxygen-dependent microbicidal response of neutrophils isolated from the peripheral blood of adult cattle [7]. In vitro conditions, *M. bovis* is also able to suppress a phytohemagglutinin-induced stimulation of bovine peripheral blood lymphocytes, however with no cytotoxic effect [8]. Similarly, other in vitro study demonstrated the ability of *M. bovis* to inhibit a Concanavalin A-induced proliferation of peripheral blood lymphocytes isolated from *M. bovis* negative donor cattle [9]. Despite so many results, the data received is still not endless, especially since it is not often possible to interpret in vitro results for in vivo conditions. Additionally, only a few studies on the characterization of the local immune response in *M. bovis* pneumonia in calves were undertaken [10,11].

To better advance our knowledge of the disease pathomechanism, an in vivo study using the experimental animal model on calves was performed which evaluated the effect of *M. bovis* on bovine peripheral blood leukocytes. To better control the *M. bovis* infection, an additional aim was to evaluate the lung local immunity of calves experimentally infected with the pathogen.

## 2. Results

Infection efficacy in the experimental calves was confirmed by clinical, post mortem and histopathologic observations and the results of immunohistochemistry (IHC) and enzyme-linked immunosorbent assay (ELISA) analyses for *M. bovis* antigen were described previously by Dudek et al. [12]. Following the calf infection with *M. bovis,* extensive caseous necrosis and lobular consolidation were observed. The *M. bovis* antigen was detected in epithelial cells of bronchioli in the lungs of all experimental calves as opposed to the controls, which were negative. All detailed post mortem results were previously described by Dudek et al. [12].

### 2.1. Hematology

**Figure 1:**
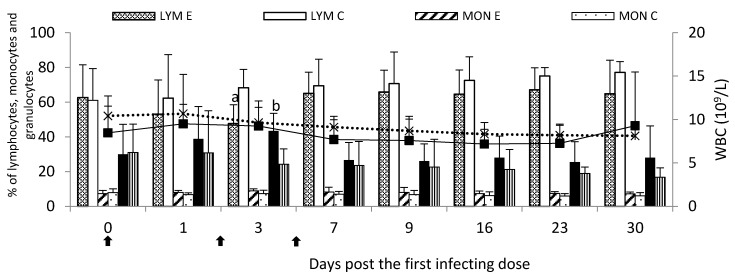
WBC count and a mean percentage of granulocytes, monocytes and lymphocytes in the peripheral blood of calves following infection with *M. bovis*. E—experimental group; C—control group; 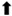
—single infecting dose of *M. bovis*; a—*p* < 0.05 between the experimental and control groups for lymphocytes; b—*p* < 0.05 between the experimental and control groups for granulocytes.

Following infection, the white blood cell (WBC) count was generally comparable to the control group throughout the study, with no significant differences (*p* < 0.05). However, the analysis of leucogram showed a comparable or lower percentage of the lymphocytes (LYM) in the experimental group throughout the study compared to the control group, with significantly lower values on Day 3 post the first infecting dose. In the experimental group, the monocyte (MON) percentage did not differ significantly (*p* < 0.05) from the control group throughout the study. However, the granulocyte (GRA) percentage was increased post the infection throughout the study compared to the control group and reached significantly (*p* < 0.05) higher values than the control group on Day 3 post the first infecting dose (Figure 1). Numerical values are presented in Figure S1.

### 2.2. Flow Cytometry

#### 2.2.1. Lymphocyte Phenotyping

**Figure 2:**
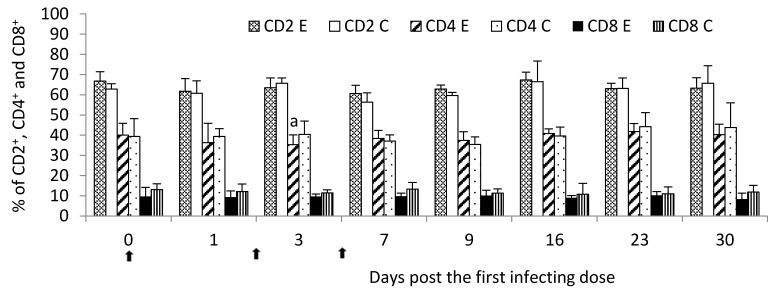
CD2^+^, CD4^+^ and CD8^+^ mean percentage in the peripheral blood of calves following infection with *M. bovis*. E—experimental group; C—control group. 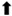
—single infecting dose of *M. bovis*; a—*p* < 0.05 between the experimental and control groups for CD4^+^.

There were no significant (*p* < 0.05) differences between the experimental and control groups in the percentage of CD2^+^, CD4^+^ and CD8^+^ cells throughout the study. However, on Day 3 post the first infecting dose, the CD4^+^ percentage was significantly lower (*p* ≤ 0.05) than the control group (Figure 2). Numerical values were presented in Supplementary Figure S2.

#### 2.2.2. Phagocytic Activity and Oxygen Metabolism of Leukocytes

**Figure 3:**
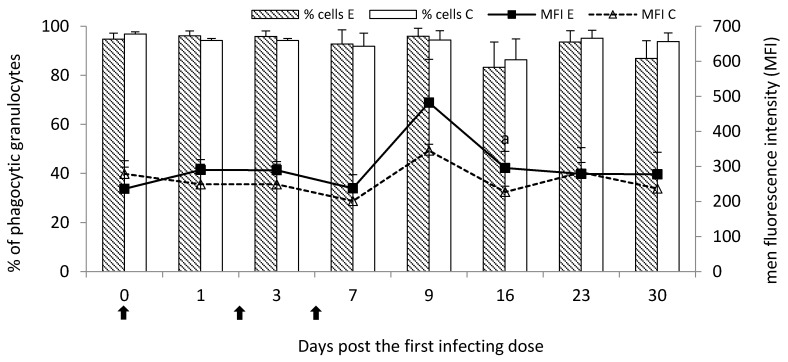
Phagocytic activity of granulocytes in the peripheral blood of calves following infection with *M. bovis* expressed as a mean percentage of phagocytic cells (bar graph) and mean fluorescence intensity (MFI; linear graph). E—experimental group; C—control group. 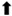
—single infecting dose of *M. bovis*; a—*p* < 0.05 between the experimental and control groups for MFI.

The percentage of phagocytic granulocytes in the peripheral blood of the experimental group did not significantly differ (*p* < 0.05) from the control group throughout the study. However, the mean fluorescence intensity (MFI) for granulocytes visibly increased on Day 9 post the first infecting dose and it was statistically significantly higher (*p* < 0.05) than the control group on Day 16 (Figure 3). Numerical values were presented in Figure S3.

**Figure 4:**
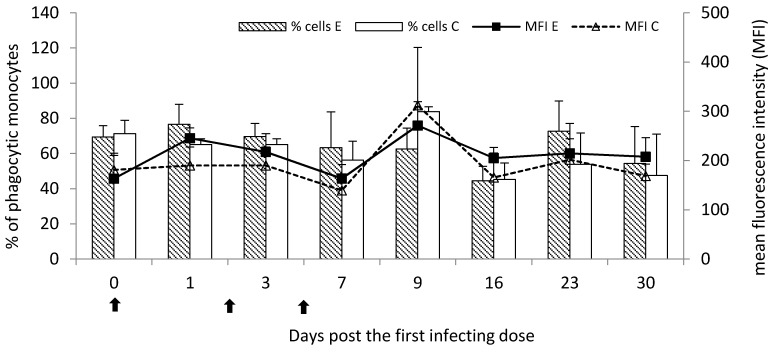
The phagocytic activity of monocytes in the peripheral blood of calves following infection with *M. bovis* expressed as a mean percentage of phagocytic cells (bar graph) and mean fluorescence intensity (MFI; linear graph). E—experimental group; C—control group. 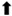
—single infecting dose of *M. bovis*.

Following the infection, the percentage of phagocytic monocytes was generally comparable to the control group throughout the study, with the exception of Day 9, when lower values were observed. However, on Day 23, a visible increase in the percentage in the experimental group was observed. Following the infection, the MFI for monocytes was generally slightly higher than the control group throughout the study, especially on Day one following the first infecting dose. However, no significant differences (*p* < 0.05) between the experimental and control groups on the phagocytic cell percentage and MFI were observed (Figure 4). Numerical values were presented in Figure S4.

**Figure 5:**
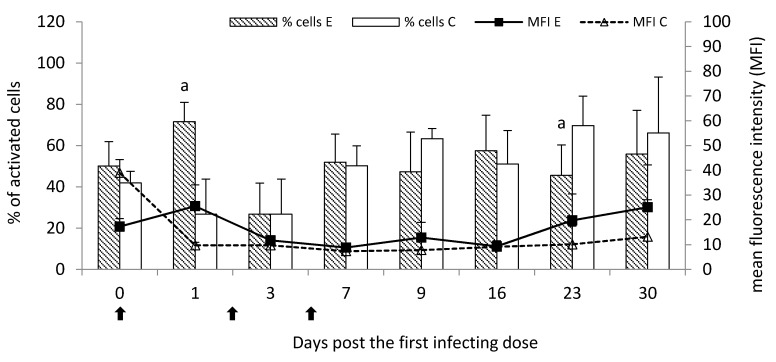
Oxygen metabolism of leukocytes in peripheral blood of calves following infection with *M. bovis* expressed as a mean percentage of cells (bar graph) and mean fluorescence intensity (MFI; linear graph) after activation by *E. coli*. E—experimental group; C—control group. 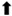
—single infecting dose of *M. bovis*. a—*p* < 0.05 between the experimental and control groups for percentage of cells.

For the oxygen metabolism, the percentage of activated leukocytes was significantly increased (*p* < 0.05) on Day one post the first infecting dose, however after that it suddenly decreased and had similar or lower values than the control group until the end of the study with significantly lower values (*p* < 0.05) on Day 23. The MFI was generally increased in the experimental group throughout the study when compared to the control group, however with no significant (*p* < 0.05) differences (Figure 5). Numerical values were presented in Figure S5.

### 2.3. Immunohistochemistry

**Figure 6:**
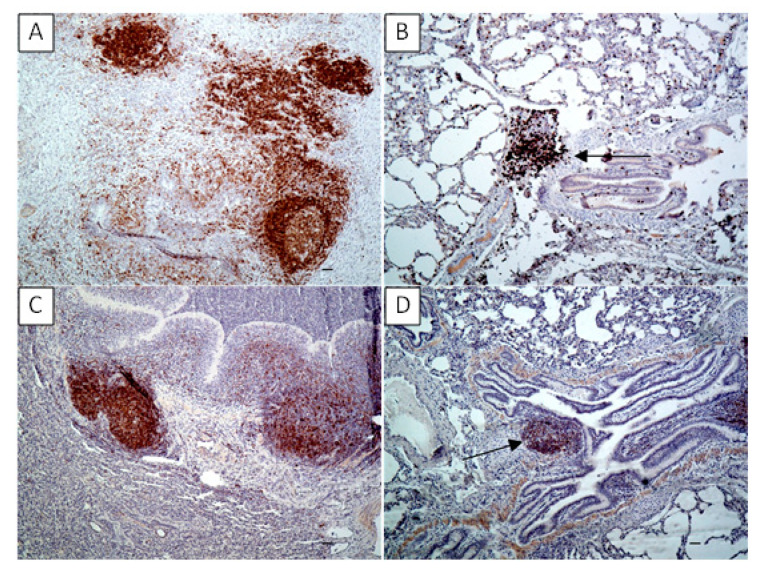
The lungs of the calves experimentally infected with *Mycoplasma bovis* (**A**,**C**), lungs of the control calves (**B**,**D**), IHC. (**A**) lung of an experimental calf. Positive immunolabeling of CD3 visible as dark brown staining in the hyperplastic bronchus-associated lymphoid tissue (BALT) and lymphoid cells scattered in the lung parenchyma. Bar = 50 µm; (**B**) lung of control calf. Positive immunolabeling of CD3 visible in BALT (arrow) and the single cells scattered in the lung parenchyma. Bar = 50 µm; (**C**) lung of an experimental calf. Positive immunolabeling of CD79a visible as dark brown staining in the hyperplastic BALT and lymphoid cells scattered in the lung parenchyma. Bar = 50 µm; (**D**) lung of control calf. Positive immunolabeling of CD79a visible in BALT (arrow). Bar = 50 µm.

In the lungs of experimentally infected calves, multiple foci of CD3 positive cells were visible in the lung parenchyma, within the hyperplastic bronchus-associated lymphoid tissue (BALT) and peribronchiolar infiltrations (Figure 6A). In the control calves, the positive reaction for the CD3 antigen was visible in BALT (Figure 6B). The positive labeling for CD79a in the infected calves was present in the hyperplastic BALT and, to a lesser extent, in the cells around bronchioli or the cells infiltrating necrotic areas (Figure 6C). In the control animals, the reaction for the CD79a antigen was visible in BALT (Figure 6D). As a result of CD3 and CD79a quantification, the immunopositive cell counts determined as mean value ± standard deviation (SD) in the control group were 1408.8 ± 88.1 for CD3 and 1437.5 ± 267 for CD79a. In the experimental group the mean cell counts for CD3 and CD79a were 3823.7 ± 1551.3 and 2118.7 ± 730.18, respectively. Compared to the controls, the experimental group displayed a significant increase (*p* < 0.05) in the number of both CD3- and CD79a-positive cells, however within the group, the mean cell count value was significantly higher (*p* < 0.05) for CD3 than for CD79a.

**Figure 7:**
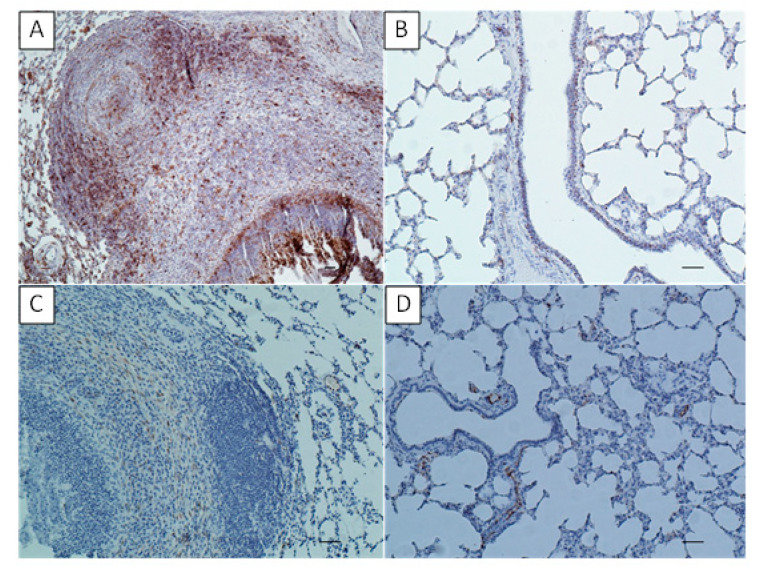
The lungs of the calves experimentally infected with *Mycoplasma bovis* (**A**,**C**), the lungs of the control calves (**B**,**D**), IHC. (**A**) lung of an experimental calf. Positive labeling of MHC class II visible as the brown staining in BALT, in the cells within the infiltrates surrounding the necrotic masses (right bottom) and in the single cells scattered in the lung parenchyma (left side). Bar = 50 µm (**B**) lung of control calf. Positive labeling of MHC class II visible as brown staining in the bronchiolar epithelial layer and the single cells around the bronchus. Bar = 50 µm; (**C**) lung of an experimental calf. Positive labeling of S100 visible as the brown staining in several cells within the granuloma. Bar = 50 µm; (**D**) lung of control calf. Positive labeling of S100 is visible in the endothelium of the blood capillaries. Bar = 50 µm.

In the lungs of the experimentally infected calves, the high concentration of MHC class II marker was found in the lymphoid cells infiltrating the granulomas, in BALT as well as in bronchiolar epithelium and the lymphoid cells in the alveolar walls (Figure 7A), while in the control animals the positive labeling for MHC class II was seen in BALT, the epithelial cells of bronchioli and in some lymphoid cells within the lung parenchyma (Figure 7B). When assessing the presence of S100 marker in the lungs of the infected calves, its high concentration was observed in vascular endothelial cells, as well as in some cells forming cellular infiltrates within granulomas (Figure 7C). In the control group, the positive IHC response was only demonstrated in vascular endothelial cells (Figure 7D).

## 3. Discussion

In the current study, the lung local immune response to *M. bovis* infection was characterized by the lymphocyte stimulation dependent on both the T- and B-cell responses, however, the most seen in the strong immunohistochemical labeling of T lymphocytes. It had a reflection in a general decrease in the percent of circulating lymphocytes, the most intensified post the second infecting dose of *M. bovis*. It was additionally confirmed by flow cytometry analysis which showed at the same time point a decline in the T-helper cell percentage. It was probably due to the migration of the lymphocytes from peripheral blood to sites of infection, including lung tissue. At the same point in time post the infection, a decrease in the percentage of circulating lymphocytes was compensated by the increased percentage of other leukocyte populations like granulocytes possibly indicating an enhancement production of these cells in the bone marrow and their release into the peripheral blood. Hermeyer et al. [11] examined the expression of CD3, CD79a, S100A8 and S100A9 markers within the lungs of the aborted bovine fetus and the newborn calf died with severe respiratory symptoms, both suffered from suppurative bronchointerstitial pneumonia due to *M. bovis* infection. The results of the study indicated the increased lymphocytic aggregates expressed CD3 and CD79a within the lung tissues of both animals confirming the presence of both T and B lymphocytes. All this suggests the activation of specific local immunity to *M. bovis* lung infection as was confirmed in the current study [11]. In another study, the identification and quantitative evaluation of CD4^+^ and CD8^+^ T lymphocytes using IHC staining in the chronic *M. bovis* pneumonia was performed. However, post the experimental infection of calves with *M. bovis,* no significant differences in the numbers of both cells in BALT of bronchioli were observed compared to the control [10].

Neutrophils and macrophages are known to be important in innate immune mechanisms in the lung, including bacteria recognizing and phagocytosis needed for the antigen presentation [4]. In the study of Hermeyer et al. [11], the increased number of macrophages expressed both S100A8 and S100A9 in the lung parenchyma of the aborted bovine fetus and neonatal calf affected with *M. bovis* was shown. Additionally, within the lung of aborted bovine fetus neutrophilic aggregates were presented [11]. In our *M. bovis* calf-infection model the increased S100 expression in the infected lungs was observed probably indicating the stimulation of phagocyte response according to Hermeyer et al. [11]. As previously proved, there is a phenomenon of *M. bovis* surviving nearby necrosis areas despite the presence of a large number of infiltrating cells like neutrophils and macrophages [13,14].

In the study of Hermeyer et al. [10] in the lungs of *M. bovis*-infected calves’ immunoreactivity of MHC class II varied dependent on the affected area. The strong MHC class II expression was revealed on the lymphoid cells in hyperplastic BALT, whereas the weak immunoreactivity or negative reaction was observed in intra-alveolar as well as perinecrotic located macrophages and in areas near caseonecrotic lesions. According to the author, such location of MHC class II expression suggest on one hand, ongoing stimulation of the lung local immunity and on the other hand downregulation of the antigen-presenting mechanisms in chronic *M. bovis* pneumonia [10]. In the current study, the high concentration of MHC class II was found in both the BALT and within the infiltrates surrounding the necrotic masses indicating general upregulation of the antigen-presenting mechanisms in response to the *M. bovis* infection. All this seems to confirm the formation of antigen-MHC class II complexes in the infected lungs, their recognition by the activated T lymphocytes and further activation of B-cell dependent response to generate specific immunity.

It is well known that granulocytes—especially neutrophils—are crucial cells in host antimicrobial defense [15]. As a predominant population of circulating leukocytes, neutrophils play an important role in the first line of cellular defense of the host against invading pathogens by various functions, including phagocytosis and oxidative burst [6,16]. In the current study, the percentage of circulating granulocytes was increased post *M. bovis* infection. It had a reflection in the slight increase in the percentage of phagocytic granulocytes at the initial stage of the disease. As the disease progresses, the visible drop in the percentage of phagocytic cells was observed, the most seen at the end of the study (a chronic stage of the disease). However, the number of phagocytosed bacteria by granulocytes on Day 16 post the first infecting dose of *M. bovis* was significantly higher than the control despite the beginning of the decline in the percentage of phagocytic cells possibly indicating increased antimicrobial activity of the cells.

Marked, however not statistically significant stimulation of phagocytic activity at the most time points post the infection was observed for circulating monocytes. Unlike granulocytes, the percentage of phagocytic monocytes was visibly increased at the chronic stage of the disease.

In the current study, the analysis of oxygen metabolism of peripheral blood leukocytes showed the initial increase in the percentage of the activated cells to different extents, the most seen post the first infecting dose of *M. bovis*. It was reflected in the visibly increased killing activity of these cells. In turn, a further decline in the activated cell percentage probably resulted from the subsequent doses of *M. bovis* and the chronic stage of the disease. It was probably in favor of mobilizing these cells within the lungs against the persisting/survival of *M. bovis* antigen. However, the killing activity of circulating leukocytes at that time was enhanced, despite the decrease in the percentage of the activated cells, possibly indicating releasing of *M. bovis* from sites of the infection, including lung tissue. In the study of Wiggins et al. [17], the effect of multiple *M. bovis* isolates (field, clinical and high passage laboratory) on Reactive Oxygen Species (ROS) production by blood leukocytes isolated from six cattle using an oxidation of dihydrorhodamine 123 (DHR-123) was measured. The leukocyte incubation with both field and clinical *M. bovis* isolates generally impaired ROS production, as opposed to the laboratory ones. In this study, the leukocyte metabolic activity using the reduction of 3-(4,5-dimethylthiazol-2-yl)-2,5-diphenyltetrazolium bromide (MTT) was also determined. Mostly following the exposure to all *M. bovis* isolates no effect on cellular metabolism of the bovine leukocytes was shown, indicating that observed suppression of ROS generation was not dependent on the leukocyte impairment of metabolic functions [17].

In the current study, using the *M. bovis* calf-infection model, the changes in the phagocytic activity and oxygen-dependent killing in the peripheral blood leukocytes was related to the stage of *M. bovis* pneumonia. However, the general stimulation of phagocytic and killing activity of circulating leukocytes in response to the *M. bovis* infection points to the upregulation of cellular antimicrobial mechanisms. The general depletion in the percent of circulating lymphocytes supporting the ongoing infection with *M. bovis*. The lung local immune response to the *M. bovis* experimental infection was characterized by the lymphocyte stimulation, the most seen in the increased T-cell response. The calf infection with *M. bovis* also caused the increased expression of the antigen-presenting cells, as well as the phagocytes further confirming the activation of lung local immune response. Despite the general stimulation of both peripheral and local cellular antimicrobial mechanisms, their effectiveness appeared insufficient in eliminating the bacteria from the host and preventing specific *M. bovis* lesions, indicating the ability of the bacteria to avoid the host immune response in *M. bovis* pneumonia.

## 4. Materials and Methods

### 4.1. Animals

Experimental study on animals was carried out in accordance with the requirements of the Local Ethics Committee on Animal Experimentation of the University of Life Sciences in Lublin, Poland (Decision no. 102/2015 admitted 8 Dec 2015), which also meet the EU standards.

The study was performed on 10 four-week-old, clinically healthy female calves housed in the institute’s vivarium. Before the proper study, the nasal swabs and blood samples were collected from the calves and examined for *Mycoplasma bovis* and other respiratory pathogens detection which was described previously by Dudek et al. [12]. After a three-week adaptive period, the calves were divided into two groups: experimental (*n* = 6) and control (*n* = 4).

All detailed information about the animals and methods used for confirmation of the infection efficacy was described previously by Dudek et al. [12].

### 4.2. Calf Challenge

The experimental calves were three times infected with 23 mL of inoculum containing the field *M. bovis* strain KP795974 suspended in sterile phosphate-buffered saline pH 7.2 (PBS), with a concentration of 1.5 × 10^8^ CFU/mL. The inoculum was prepared as described previously [18] and given three times in total; for the first time on Day 0 of the study and then two times at 48 h intervals; twice intratracheally and once by a nasal aerosol application. Instead, the control animals were administered with sterile PBS. It was described previously in detail by Dudek et al. [12].

### 4.3. Sample Collection

Blood samples were collected in EDTA tubes (for hematology and CD marker detection) or standard heparinized tubes (concerning analyses of phagocytic activity and oxygen metabolism of leukocytes) on Days 0, 1, 3, 7, 9, 16, 23 and 30 post the first *M. bovis* infecting dose. At the end of the experiment on Day 30, all experimental and two control calves were euthanized to collect the lung samples for pathologic and immunohistochemical analyses.

### 4.4. Hematology

White blood cell (WBC) count and percentage of lymphocytes, monocytes and granulocytes were calculated in an automatic veterinary blood analyzer (Exigo, Boule Medical AB, Spånga, Sweden).

### 4.5. Flow Cytometry

#### 4.5.1. Lymphocyte Phenotyping

Peripheral blood lymphocyte analysis using the CD markers for T-cells (CD2^+^), T-helper cells (CD4^+^) and T-cytotoxic suppressor cells (CD8^+^) was performed by a flow cytometer (Coulter Epics XL 4C, Beckman Coulter Company, Brea, CA, USA) according to the method described previously by Dudek et al. [18].

#### 4.5.2. Phagocytic Activity and Oxygen Metabolism of Leukocytes

Phagocytic activity and oxygen metabolism of peripheral blood leukocytes were evaluated according to the manufacturer’s instructions of two separated commercial kits: Phagotest™ for leukocyte phagocytic activity and Phagoburst™ for oxygen metabolism analysis, both manufactured by Glycotope Biotechnology GmbH (Heidelberg, Germany) and analyzed using Epics XL flow cytometer (Beckman Coulter, Miami, FL, USA). The granulocyte and monocyte phagocytic activity was expressed as the percentage of cells that engulfed bacteria as well as mean fluorescence intensity (MFI) of the cells for estimating of phagocytosed bacteria amount. The oxygen metabolism of peripheral blood leukocytes was determined as the percentage of cells activated by *E. coli* as well as MFI for the measurement of phagocytic activity of leukocytes.

### 4.6. Immunohistochemistry

The collected lung samples were examined using an immunohistochemical staining for the detection of local immune response markers such as CD3 (T-cells), CD79 (B-cells), MHC class II and S100. Previously prepared sections were deparaffinized in xylene, rehydrated in descending ethanol concentrations, then incubated in 3% H_2_O_2_ diluted in methanol for 10 min and submitted to heat-induced epitope retrieval in citrate buffer (pH 6.0) using a pressure cooker for 20 min. Depending on the marker the slides were then incubated for one hour with primary antibodies as follows: rabbit anti-CD3 monoclonal antibody (A045201, DAKO, Glostrup, Denmark) at dilution 1:100 for CD3 detection; mouse monoclonal anti-CD79a antibody [HM57] (ab62650, Abcam, Cambridge, UK) at dilution 1:400 for CD79a detection; mouse anti-HLA-DR Antigen, Alpha-Chain, Clone TAL.1B5 (M074601-2, DAKO, Glostrup, Denmark) at dilution 1:40 for MHC class II detection and FLEX Polyclonal Rb anti-S100, RTU (GA50461-2, DAKO) at dilution 1:1 for S100 detection. The antibody detection was performed using the Dako REAL EnVision Detection System, Peroxidase/DAB, Rabbit/Mouse (K5007, DAKO, Glostrup, Denmark), involving an incubation with a peroxidase-conjugated polymer as a secondary antibody (for 30 min) and DAB^+^ Chromogen applied for a visualization of the reaction. Sections were counterstained with Mayer’s hematoxylin, dehydrated and mounted. Sections incubated with PBS instead of the primary antibody were used to confirm the specificity of the staining. The tissues were analyzed under a light microscope (Axiolab, Zeiss, Oberkochen, Germany) for the presence of brown staining indicating positive labeling of *M. bovis*, CD3, CD79a, MHC class II and S100. To determine the difference between the number of T- and B-lymphocytes infiltrating the tissue in the examined sections of experimental group and to compare number of the two cell–type populations between the experimental and control groups, the CD3- and CD79a-positive cells were counted in 20 high power fields (400x) comprising the cell infiltrations and/or BALT in each slide.

### 4.7. Statistical Analysis

The results are presented as arithmetic means or mean percentage ± standard deviation. The differences between the mean values recorded in the E and C groups at the same time point were analyzed using *t*-test with a statistically significant level of *p* < 0.05. The same test and the *p*-value were applied to determine the difference between the mean values of summarized cell counts for the CD3 and CD79a markers analyzed with IHC in the experimental and control groups.

## Supplementary Materials

The following are available online at https://www.mdpi.com/2076-0817/9/5/407/s1, Figure S1. Numerical values for hematology; Figure S2. Numerical values for lymphocyte phenotyping; Figure S3. Numerical values for phagocytic activity of granulocytes; Figure S4. Numerical values for phagocytic activity of monocytes; Figure S5. Numerical values for oxygen metabolism of leukocytes.
