# Supplementary material for: Analysis of the Leukocyte Response in Calves Suffered from Mycoplasma bovis Pneumonia
Source: Pathogens. 2020 May 24;9(5):407. doi: 10.3390/pathogens9050407 (PMC7281192; doi:10.3390/pathogens9050407)
Supplement: Supplementary file 1 [file pathogens-09-00407-s001.pdf]

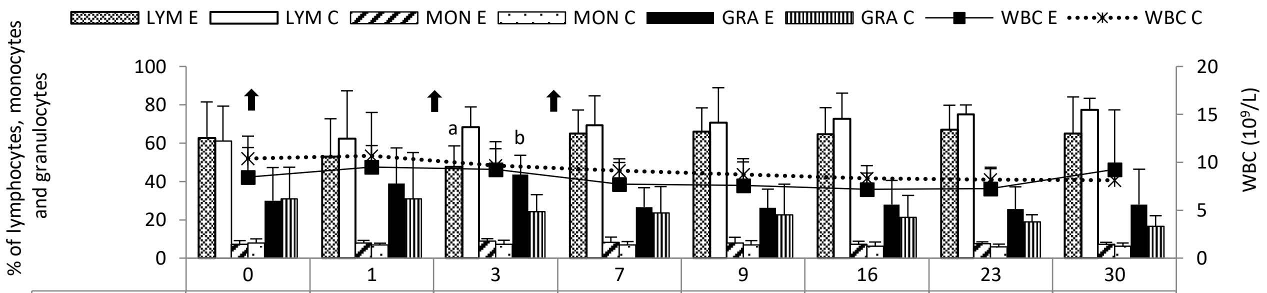

	0	1	3	7	9	16	23	30
LYM E	62.67	53.12	47.68	65.07	65.83	64.66	67.08	64.82
LYM C	61.08	62.38	68.35	69.45	70.63	72.55	75.05	77.18
MON E	7.32	7.98	8.95	8.25	8.03	7.32	7.42	7.16
MON C	7.93	6.78	7.35	7.03	6.73	6.20	6.00	6.10
GRA E	30.02	38.90	43.45	26.68	26.13	28.02	25.50	28.02
GRA C	31.00	30.85	24.30	23.53	22.65	21.25	18.95	16.73
WBC E	8.47	9.50	9.25	7.70	7.58	7.18	7.26	9.28
WBC C	10.43	10.68	9.65	9.13	8.70	8.33	8.20	8.13

Days post the first infecting dose

Supplementary Figure S1. Numerical values for haematology.

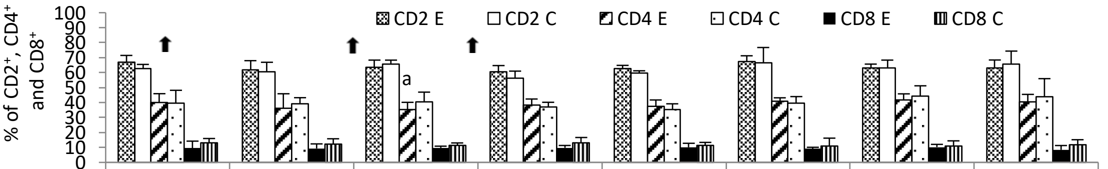

	0	1	3	7	9	16	23	30
CD2 E	66.84	61.73	63.46	60.61	62.83	67.39	63.06	63.23
CD2 C	62.85	60.73	65.72	56.36	59.68	66.48	63.14	65.74
CD4 E	40.08	36.26	35.35	38.41	37.43	40.79	41.78	40.30
CD4 C	39.39	39.35	40.41	37.11	35.41	39.58	44.25	43.80
CD8 E	9.62	9.35	9.72	9.77	10.09	9.03	10.24	8.38
CD8 C	13.06	12.11	11.45	13.28	11.29	10.81	10.99	11.83

Days post the first infecting dose

Supplementary Figure S2. Numerical values for lymphocyte phenotyping.

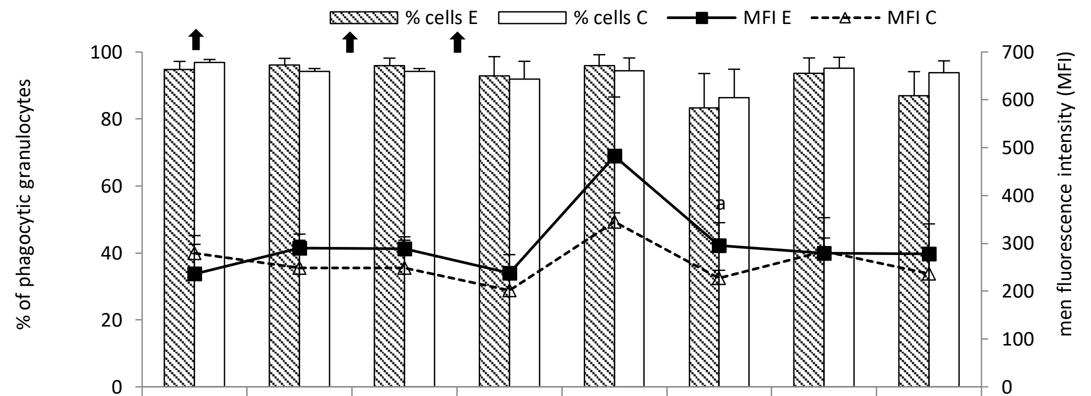

	0	1	3	7	9	16	23	30
% cells E	94.76	96.10	95.90	92.83	96.00	83.25	93.62	86.92
% cells C	96.83	94.23	94.23	91.93	94.47	86.33	95.13	93.83
MFI E	235.97	290.07	289.40	237.80	482.20	295.76	278.82	277.76
MFI C	278.90	249.23	249.23	201.58	345.07	227.10	283.43	237.13

Days post the first infecting dose

Supplementary Figure S3. Numerical values for phagocytic activity of granulocytes.

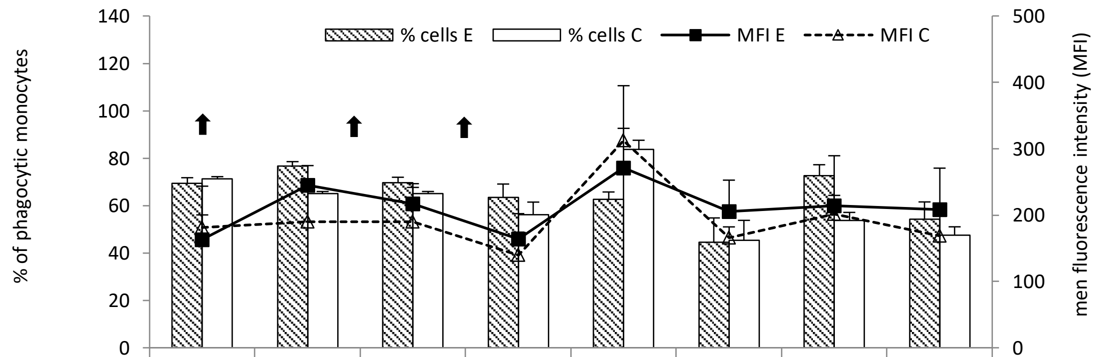

	0	1	3	7	9	16	23	30
% cells E	69.32	76.53	69.67	63.30	62.53	44.45	72.62	54.32
% cells C	71.27	65.10	65.10	56.18	83.80	45.23	53.83	47.50
MFI E	163.22	245.30	217.37	163.52	271.20	205.10	214.44	207.80
MFI C	181.20	190.10	190.10	139.45	312.13	165.63	201.85	168.83

Days post the first infecting dose

Supplementary Figure S4. Numerical values for phagocytic activity of monocytes.

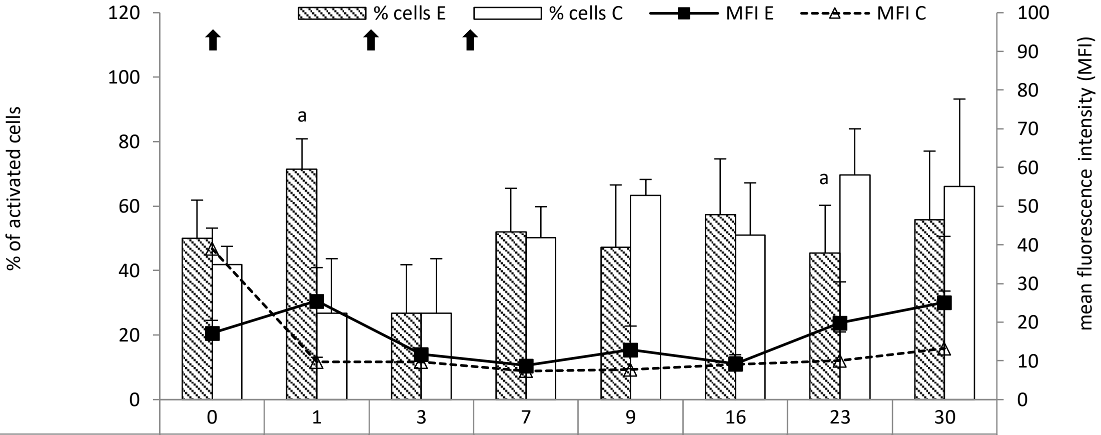

	0	1	3	7	9	16	23	30
% cells E	50.03	71.53	26.78	51.93	47.27	57.44	45.52	55.82
% cells C	41.87	26.70	26.70	50.20	63.30	51.03	69.68	66.10
MFI E	17.27	25.53	11.73	8.84	12.86	9.31	19.81	25.06
MFI C	39.00	9.74	9.74	7.37	7.82	9.15	10.13	13.19

Supplementary Figure S5. Numerical values for oxygen metabolism of leukocytes.
